# Evaluation of cesarean delivery rates and factors associated with cesarean delivery among women enrolled in a pregnancy cohort study at two tertiary hospitals in Thailand

**DOI:** 10.1186/s12884-024-06314-4

**Published:** 2024-02-21

**Authors:** Orada Patamasingh Na Ayudhaya, Wanitchaya Kittikraisak, Podjanee Phadungkiatwatana, Danielle Rentz Hunt, Krissada Tomyabatra, Tawee Chotpitayasunondh, Romeo R. Galang, Karen Chang, Tana Brummer, Lunthaporn Puttanavijarn, Parker Malek, Fatimah S. Dawood, Joshua A. Mott, for the PRIME Study Group, Surasak Kaoiean, Suvanna Asavapiriyanont, Nattinee Srisantiroj, Boonsong Rawangban, Sirichat Rongsak, Wiboon Kanjanapattanakul, Piyarat Suntarattiwong, Bajaeee Chotpitayasunondh, Chalinthorn Sinthuwattanawibool, Waraporn Sakornjun, Damon Ellison, Chonticha Klungthong, Kittinun Hussem, Stefan Fernandez, Louis Macareo, Meredith G. Wesley, Eduardo Azziz-Baumgartner, Danielle Hombroek

**Affiliations:** 1 Nopparat Rajathanee Hospital, Ministry of Public Health, Bangkok, Thailand; 2 Influenza Program, Thailand Ministry of Public Health - U.S. Centers for Disease Control and Prevention Collaboration, Ministry of Public Health (DDC building 7), Tiwanon Road, Nonthaburi, 11000 Thailand; 3 Rajavithi Hospital, Ministry of Public Health, Bangkok, Thailand; 4 Abt Associates, Atlanta, GA USA; 5 Queen Sirikit National Institute of Child Health, Ministry of Public Health, Bangkok, Thailand; 6 Division of Reproductive Health, U.S. Centers for Disease Control and Prevention, Atlanta, GA USA; 7 Influenza Division, U.S. Centers for Disease Control and Prevention, Atlanta, GA USA

**Keywords:** Cesarean delivery, Pregnant woman, Robson Classification

## Abstract

**Background:**

Cesarean delivery rates have increased globally resulting in a public health concern. We estimate rates of cesarean deliveries among Thai women using the World Health Organization (WHO) Robson Classification system and compare rates by Robson group to the Robson guideline for acceptable rates to identify groups that might benefit most from interventions for rate reduction.

**Methods:**

In 2017 and 2018, we established cohorts of pregnant women aged ≥ 18 years seeking prenatal care at two tertiary Thai hospitals and followed them until 6–8 weeks postpartum. Three in-person interviews (enrollment, end of pregnancy, and postpartum) were conducted using structured questionnaires to obtain demographic characteristics, health history, and delivery information. Cesarean delivery indication was classified based on core obstetric variables (parity, previous cesarean delivery, number of fetuses, fetal presentation, gestational week, and onset of labor) assigned to 10 groups according to the Robson Classification. Logistic regression was used to identify factors associated with cesarean delivery among nulliparous women with singleton, cephalic, term pregnancies.

**Results:**

Of 2,137 participants, 970 (45%) had cesarean deliveries. The median maternal age at delivery was 29 years (interquartile range, 25–35); 271 (13%) participants had existing medical conditions; and 446 (21%) had pregnancy complications. The cesarean delivery rate varied by Robson group. Multiparous women with > 1 previous uterine scar, with a single cephalic pregnancy, ≥ 37 weeks gestation (group 5) contributed the most (14%) to the overall cesarean rate, whereas those with a single pregnancy with a transverse or oblique lie, including women with previous uterine scars (group 9) contributed the least (< 1%). Factors independently associated with cesarean delivery included age ≥ 25 years, pre-pregnancy obesity, new/worsen medical condition during pregnancy, fetal distress, abnormal labor, infant size for gestational age ≥ 50^th^ percentiles, and self-pay for delivery fees. Women with existing blood conditions were less likely to have cesarean delivery.

**Conclusions:**

Almost one in two pregnancies among women in our cohorts resulted in cesarean deliveries. Compared to WHO guidelines, cesarean delivery rates were elevated in selected Robson groups indicating that tailored interventions to minimize non-clinically indicated cesarean delivery for specific groups of pregnancies may be warranted.

**Supplementary Information:**

The online version contains supplementary material available at 10.1186/s12884-024-06314-4.

## Introduction

World Health Organization (WHO) suggests that the cesarean delivery rate should be at 10–15% and based on clinical indications, but the global rate has been increasing since the 1970s [1]. Globally > 21% of all childbirths during 2010–2018 were born by cesarean delivery; this number is projected to rise with 29% of all births likely to take place by caesarean delivery by 2030 [2]. Clinically indicated cesarean delivery may improve maternal and fetal outcomes and reduce morbidity and mortality [3]. However, unnecessary cesarean delivery procedure may lead to higher hospital cost, longer hospital stay, and increased maternal and fetal morbidity and mortality [4, 5]. At a population level, cesarean delivery rates higher than 10% are not associated with reductions in maternal and newborn mortality rates [4]. Additionally, the cesarean delivery procedure can cause significant and sometimes permanent complications, disabilities or death. Risks from cesarean delivery to women include intra/post-operative hemorrhage, wound infection, deep vein thrombosis and adverse outcomes for future pregnancy [6–10]. Additionally, study has shown that children born by cesarean delivery had increased risk of obesity and asthma up to the age of 5 and 12 years, respectively [10].

In Thailand, the cesarean delivery rate increased from 11% in 1992 to 24% in 2011 in both public and private hospitals [11]. Another report in 2022 indicated an averaged cesarean delivery rate of 42% across 12 health regions excluding Bangkok (range 32–50%) [12]. While the rate in Bangkok has not been formally established, report from a large tertiary public hospital in Bangkok indicated a rate of 49% among nearly 5,000 deliveries in 2017 [13]. Like elsewhere, possible causes of the rise in cesarean delivery rates in Thailand may include advanced maternal age, increased detection of fetal distress during labor from wider use of electronic fetal monitoring, and non-medical factors such as a desire to schedule delivery [14, 15]. In 2019, the Ministry of Public Health (MOPH) and the Royal Thai College of Obstetricians and Gynecologists developed a policy to reduce the cesarean delivery rate throughout Thailand [16]. As part of this policy, the Thai MOPH recommends the use of the 10-Group classification (the “Robson Classification”) as a tool to determine cesarean delivery indication [17]. The Robson Classification is a global standard to identify groups of pregnant women contributing most and least to overall cesarean delivery rate, assess the effectiveness of interventions targeted at optimizing appropriate use of cesarean delivery while minimizing non-clinically indicated cesarean deliveries, and assess the quality of care and clinical management practices. The Robson Classification can be used to compare cesarean delivery rates in the same setting prospectively and between different settings [3, 18, 19]. When cesarean delivery rates are higher than expected, the WHO recommends focusing efforts at rate reduction in nulliparous women with singleton, cephalic, term pregnancies (Robson group 1). Additionally, the trial of vaginal birth after cesarean delivery (VBAC) has also been recommended [20] in women with previous cesarean delivery to further reduce the cesarean delivery rates, although VBAC is not officially endorsed or widely practiced in Thailand [21, 22].

As part of a larger prospective longitudinal cohort study assessing the effect of influenza virus infection during pregnancy on pregnancy and perinatal outcomes [23, 24], we conducted an analysis of data from participants who delivered at two hospitals in Thailand to estimate rates of cesarean deliveries and characterize them using the Robson Classification. Additionally, to inform efforts for cesarean delivery rate reduction, we examined factors associated with cesarean delivery among nulliparous women with singleton, cephalic, term pregnancies (Robson group 1).

## Methods

### Setting and participant enrollment

This study was conducted at two tertiary care hospitals in Thailand: Rajavithi Hospital and Nopparat Rajathanee Hospital. Rajavithi Hospital mostly serves the population living in the central part of Bangkok while Nopparat Rajathanee Hospital serves the northeastern part of the city. During this study, each hospital delivered approximately 6,000 babies per year. A detailed description of the cohorts and study protocol was previously published [23]. Briefly, in 2017 and 2018 we established cohorts of pregnant women aged ≥ 18 years seeking antenatal care (ANC) at the study hospitals and followed them until 6–8 weeks postpartum.

### The Robson Classification

The Robson Classification system classifies all deliveries into 10 mutually exclusive groups based on obstetric parameters: parity, previous cesarean delivery, number of fetus, fetal presentation, gestational week, and onset of labor. Detail of the Robson Classification is published in the WHO Robson Classification Implementation Manual [1].

### Prenatal care services and mode of delivery

Thailand provides free of charge ANC services to all Thai pregnant women insured by the government-provided health insurance schemes and to foreign individuals living in Thailand who self-purchase health insurance. The standard ANC includes 5–8 visits (at approximately 12, 18, 26, 32, 34, 36, 38, and 40 weeks of pregnancy) and delivery service. During the ANC visits, physical examination, health education, laboratory testing, and at least one ultrasound to estimate delivery date and to screen for fetal anomalies are conducted. An additional ultrasound is performed during the third trimester only in selected women to determine fetal presentation or if there is a concern of intrauterine growth restriction. Mode of delivery is generally decided by obstetricians. If clinically indicated (e.g., previous cesarean delivery, fetal distress, fetal malpresentation, placenta previa, certain types of medical conditions), a cesarean delivery will be scheduled and paid for by the health insurance scheme. Women without a clinical indication for cesarean delivery may elect to have one based on personal preference. In such cases, women pay the related delivery fees as it is deemed by the health insurance schemes as an unnecessary procedure.

### Data collection

We conducted three in-person interviews with study participants using structured questionnaires. The enrollment interview collected information about demographic, socio-economic, and clinical characteristics. The end of pregnancy interview, conducted within seven days after delivery, collected information about pregnancy complications, prenatal care, health behaviors since enrollment, mode of delivery, pregnancy outcomes, and length of the peripartum hospital stay. The postpartum interview, conducted 6–8 weeks after delivery, collected information about maternal postpartum and neonatal clinical courses. When available, we reviewed medical records to confirm participants’ prenatal care, pregnancy course, and delivery/perinatal outcomes.

### Data analysis

For analytic purposes, only participants with gestational weeks at delivery and mode of delivery information were included. We conducted descriptive analyses to characterize participants’ demographic, socio-economic, and clinical characteristics, comparing those with cesarean versus vaginal deliveries. Length of neonatal hospital stay was calculated from birth to discharge and compared between those born to mothers who had cesarean delivery and the mothers with vaginal delivery using the two-sample Wilcoxson rank-sum test. The indication of cesarean delivery was classified based on core obstetric variables assigned to 10 groups as suggested by Robson [17]. The following outcomes based on the WHO Robson Classification Implementation Manual [1] were expressed as percentages: size of each Robson group (number of women in each category divided by the total number of women), cesarean delivery rate by category (number of cesarean deliveries in the category divided by the number of women in the category), and contribution of each category to the overall cesarean delivery rate (number of cesarean deliveries in each category divided by the total number of cesarean deliveries). To characterize the study population in comparison to reference populations used to develop the Robson categories, we compared the size of each Robson group in the study with the reference distribution provided in the WHO Robson Classification Implementation Manual. To identify Robson categories with higher than recommended or expected cesarean delivery rates, we compared the cesarean delivery rate by category in our study to target rates reported in the manual.

With the aim to inform efforts at cesarean delivery rate reduction, we restricted analyses of factors associated with cesarean delivery to nulliparous women with singleton, cephalic, term pregnancies. We used logistic regression to compare the following characteristics among women with vaginal versus cesarean deliveries: participants’ characteristics (age, nationality, marital status, employment, education, pre-pregnancy body mass index [BMI, kg/m^2^], insurance type, monthly household income, residence location, use of cigarette and alcohol, and psychological stressor score [based on 17 questions]); delivery facility; enrollment year; prenatal care history (number of visits, trimester at first visit); illness history (type and number of existing chronic conditions, type of new or worsened conditions during pregnancy, hospitalization history during pregnancy); complications during the current pregnancy (type and number of complications, trimester at diagnosis; Supplementary Table 1); and baby weight at delivery. Variables with *p*-value ≤ 0.20 in bivariate analyses were entered into a multivariable logistic regression model. We used stepwise backward elimination approach to construct the final model with variables with *p*-values of < 0.05 retained. The first order 2- and 3-way interactions between variables in the final model were investigated. All data were analyzed using Stata, version 16 (Stata Corp., College Station, Texas, USA), with a two-tailed *p*-value of < 0.05 considered statistically significant.

## Results

### Characteristics of study participants

A total of 2,810 participants were enrolled during 2017 and 2018. Overall, 2,137 (76%) had the gestational weeks at delivery and mode of delivery information: 970 (45%) with cesarean delivery, and 1,167 (55%) with vaginal delivery. Six (< 1%) participants used assisted fertilization technologies to become pregnant with their current pregnancies. Most participants (1,759, 82%) were Thai, 243 (11%) were Burmese, 92 (4%) were Cambodian, 41 (2%) were Laotian, and 2 (< 1%) were Vietnamese. The median maternal age at delivery was 29 years (interquartile range [IQR], 25–35). Two hundred and forty-five (11%) participants had existing chronic medical conditions, and 446 (21%) had complications during the current pregnancies (Supplementary Table 1).

Baseline and pregnancy characteristics among women with and without gestational weeks at delivery and mode of delivery information are shown in Supplementary Table 2. A high proportion of women without gestational weeks at delivery and/or mode of delivery information had missing data for selected characteristics precluding a valid comparison of these characteristics among women included and excluded from the analysis. Among women with information on selected characteristics of interest, those without gestational weeks at delivery and/or mode of delivery information were more likely than those with gestational weeks at delivery and mode of delivery information to have a lower educational level, lower monthly household income, a pre-pregnancy BMI categorized as underweight, and normal infant weight at delivery. Women without gestational weeks at delivery and/or mode of delivery information also were less likely to have previous uterine scars indicative of previous cesarean delivery, spontaneous labor, and a plan for cesarean delivery in advance.

Among participants with gestational weeks at delivery and modes of delivery information, participants with cesarean versus vaginal deliveries differed by age, highest educational level, monthly household income, health insurance type, pre-pregnancy BMI, trimester of 1^st^ ANC visit, number of complications during the current pregnancy, fetal weight at delivery, and spontaneous labor (Table 1). Babies born by cesarean delivery had a longer hospital stay than those born by vaginal delivery (median 4 days, IQR 3-4 days versus median 3 days, IQR 2-4 days, *p*-value <0.01).

**Table 1:** Characteristics of women with gestational weeks at delivery and mode of delivery information (number [%])

Characteristic	All participants (*N* = 2137)	Nulliparous women with singleton, cephalic, term pregnancies			
		**All delivery types (*N* = 815)**	**Cesarean delivery (*N* = 365)**	**Vaginal delivery (*N* = 450)**	***P*-value**^**a**^
**Age at delivery (years)**					< 0.01
< 25	513 (24)	297 (36)	80 (22)	217 (48)	
25–29	557 (26)	231 (28)	107 (29)	124 (28)	
30–34	530 (25)	162 (20)	91 (25)	71 (16)	
≥ 35	537 (25)	125 (15)	87 (24)	38 (8)	
**Marital status**					0.92
Married or cohabitating	2066 (97)	783 (96)	352 (96)	431 (96)	
Single, divorced, widowed, separated	69 (3)	31 (4)	13 (4)	18 (4)	
Unknown	2 (< 1)	1 (< 1)	0 (0)	1 (< 1)	
**Highest year of school completed**					< 0.01
None	61 (3)	19 (2)	6 (2)	13 (3)	
Primary school	771 (36)	206 (25)	81 (22)	125 (28)	
Secondary	533 (25)	211 (26)	81 (22)	130 (29)	
Post-secondary/university	765 (36)	375 (46)	195 (55)	180 (40)	
Unknown	7 (< 1)	4 (< 1)	2 (1)	2 (< 1)	
**Monthly household income (Baht)**					< 0.01
< 20,000	573 (27)	213 (26)	73 (20)	140 (31)	
20,000–39999	993 (46)	353 (43)	156 (43)	197 (44)	
40,000–49999	231 (11)	96 (12)	44 (12)	52 (12)	
≥ 50,000	336 (16)	152 (19)	91 (25)	61 (14)	
Unknown	4 (< 1)	1 (< 1)	1 (< 1)	0 (0)	
**Type of health insurance used for delivery**					< 0.01
Social Security Scheme	1029 (48)	413 (51)	185 (51)	228 (51)	
Universal Coverage Scheme	398 (19)	135 (17)	44 (12)	91 (20)	
Civil Servant Medical Benefit Scheme (including state enterprises’)	109 (5)	49 (6)	33 (9)	16 (4)	
Health card	226 (11)	83 (10)	34 (9)	49 (11)	
No insurance (self-pay)	342 (16)	121 (15)	65 (18)	56 (12)	
Others^b^	31 (1)	12 (1)	3 (1)	9 (2)	
Unknown	2 (< 1)	2 (< 1)	1 (< 1)	1 (< 1)	
**Pre-pregnancy body mass index (kg/m**^**2**^**)**^**c**^					< 0.01
< 18.5 (underweight)	319 (15)	148 (18)	46 (13)	102 (23)	
18.5–24.9 (normal)	1271 (59)	506 (62)	221 (61)	285 (63)	
25–29.9 (overweight)	336 (16)	98 (12)	52 (14)	46 (10)	
≥ 30 (obese)	185 (9)	52 (6)	38 (10)	14 (3)	
Unknown	26 (1)	11 (1)	8 (2)	3 (1)	
**Parity**^**c**^					n/a
0	930 (44)	815 (100)	365 (100)	450 (100)	
≥ 1	1207 (56)	0 (0)	0 (0)	0 (0)	
**Self-reported number of current chronic medical conditions**^**d**^					0.77
0	1866 (87)	730 (90)	325 (89)	405 (90)	
1	245 (11)	80 (10)	37 (10)	43 (9)	
> 1	26 (1)	5 (1)	3 (1)	2 (< 1)	
**New medical condition during the current pregnancy**^**c,**^^**d**^					< 0.01
Yes	43 (2)	12 (3)	11 (3)	1 (< 1)	
No	2094 (98)	803 (97)	354 (97)	449 (100)	
**Worsening chronic medical condition during the current pregnancy**^**c,**^^**d**^					0.70
Yes	14 (1)	2 (< 1)	1 (< 1)	1 (< 1)	
No	2123 (99)	813 (100)	364 (100)	449 (100)	
**Psychosocial stressor**^**e**^					0.11
Yes	336 (16)	133 (16)	51 (14)	82 (18)	
No	1799 (84)	681 (84)	314 (86)	367 (82)	
Unknown	2 (< 1)	1 (< 1)	0 (0)	1 (< 1)	
**Previous uterine scar**^**c**^					n/a
Yes	343 (16)	0 (0)	0 (0)	0 (0)	
No	1794 (84)	815 (100)	365 (100)	450 (100)	
**Multiparous**^**c**^					n/a
Yes	1351 (37)	0 (0)	0 (0)	0 (0)	
No	786 (63)	815 (100)	365 (100)	450 (100)	
**Number of prenatal care visits before delivery**^**c**^					0.42
1–3	43 (2)	16 (2)	8 (2)	8 (2)	
4–6	170 (8)	41 (5)	17 (5)	24 (5)	
7–9	775 (36)	272 (33)	114 (31)	158 (35)	
> 9	1136 (53)	480 (59)	225 (62)	255 (57)	
Unknown	13 (1)	6 (1)	1 (< 1)	5 (1)	
**Trimester of 1st prenatal care visit**^**c**^					0.04
First	1477 (70)	600 (74)	284 (78)	316 (70)	
Second	622 (29)	202 (25)	78 (21)	124 (28)	
Third	34 (2)	10 (1)	3 (1)	7 (2)	
Unknown	4 (< 1)	3 (< 1)	0 (0)	3 (1)	
**Number of complications during this pregnancy**^**c,**^^**f**^					< 0.01
0	1691 (79)	654 (80)	264 (72)	390 (87)	
1	392 (18)	141 (17)	87 (24)	54 (12)	
> 1	54 (3)	20 (2)	14 (4)	6 (1)	
**Infant weight at delivery**^**c**^					< 0.01
Small for gestational age^g^	194 (9)	98 (12)	28 (8)	70 (16)	
Normal	1769 (83)	670 (82)	300 (82)	370 (82)	
Large for gestational age^g^	174 (8)	47 (6)	37 (10)	10 (2)	
**Presentation**^**c**^					n/a
Cephalic	2040 (95)	815 (100)	365 (100)	450 (100)	
Breech	68 (3)	0 (0)	0 (0)	0 (0)	
Transverse	5 (< 1)	0 (0)	0 (0)	0 (0)	
Other malpresentation	24 (1)	0 (0)	0 (0)	0 (0)	
**Onset of labor**^**c**^					< 0.01
Spontaneous	1509 (71)	522 (64)	288 (79)	234 (52)	
Induction^h^	628 (29)	293 (36)	77 (21)	216 (48)	
**Cesarean delivery planned**					< 0.01
Yes	807 (38)	348 (43)	348 (95)	0 (0)	
No	1330 (62)	467 (57)	17 (5)	450 (100)	
**Gestational weeks at delivery**^**c**^					0.34
< 37	199 (9)	0 (0)	0 (0)	0 (0)	
37–38	875 (41)	319 (39)	151 (41)	168 (37)	
39–40	995 (47)	458 (56)	195 (53)	263 (58)	
≥ 41	68 (3)	38 (5)	19 (5)	19 (4)	
**Length of hospital stay (days)**^**c**^					< 0.01
< 3	458 (21)	188 (23)	9 (2)	179 (40)	
3	820 (38)	301 (37)	142 (39)	159 (35)	
4–6	639 (30)	262 (32)	175 (48)	87 (19)	
7–13	91 (4)	33 (4)	21 (6)	12 (3)	
≥ 14	52 (2)	9 (1)	5 (1)	4 (1)	
Unknown	77 (4)	22 (3)	13 (4)	9 (2)	

^a^Comparing nulliparous participants with singleton, cephalic, term pregnancies who had cesarean versus vaginal deliveries

^b^Private health insurance, handicap card, and others

^c^Information abstracted from medical records

^d^Questions asking about the following conditions: respiratory/lung problem; heart disease or heart condition; endocrine disorder such as thyroid problem or diabetes; blood problem such as sickle cell disease, or thalassemia; kidney or bladder disease; hepatitis, jaundice or liver disease, excluding hepatitis A and E; problem with the immune system, excluding HIV; cancer; neurologic or neuromuscular disorder; HIV infection; mental health condition such as anxiety or depression; and others

^e^Based on 17 questions about major events or changes that may have happened to one’s life

^f^Questions asking about the following conditions: gestational diabetes, gestational hypertension, oligohydramnios, placenta previa, heavy uterine bleeding, severe anemia, polyhydramnios, pre-eclampsia, eclampsia, incompetent cervix or cervical insufficiency, intrauterine growth restriction, multiple myoma uteri, anterior vaginal cyst, uterine fibrosis, ovarian cyst, threatened abortion, premature rupture of membrane, urinary tract infection, chickenpox, anti-E positive, twin to twin transfusion, fetal beta thalassemia/hemoglobin E disease, and dead fetus in utero at 36 weeks

^g^Small for gestational age was defined as birth weight < 10% of the same gestational age and sex in the same population; large for gestational age was defined as birth weight > 90% of the same gestational age and sex in the same population

^h^All women with gestational weeks > 40 were induced

### Robson Classification

The overall cesarean delivery rates and the Robson Classification distribution were similar between the study hospitals and years; therefore, the data were combined into a single dataset. Compared to reference population sizes in the WHO Robson Classification Implementation Manual, the size of the obstetric population in each Robson group in this study was similar, except groups 3 and 4 which were larger in the study (30% in the WHO reference population versus 41% in the study; Table 2). The overall rate of cesarean delivery was 45% (970/2,137), with the highest rates in groups 5–10 (91–100%) and the lowest rate in group 4 (14%). Compared to the Robson guideline for achievable or acceptable rates in each Robson group, cesarean delivery rates were higher in Robson group 1 (< 10% recommended rate versus 54% in this study), group 3 (~ 3% recommended rate versus 29% in this study), group 5 (50–60% recommended rate versus 98% in this study), group 8 (~ 60% recommended rate versus 91% in this study), and group 10 (~ 30% recommended rate versus 46% in this study). Participants in Robson group 5 contributed the most (14%) to the overall cesarean rate and those in group 9 contributed the least (< 1%).

**Table 2:** Robson Classification among women with gestational weeks at delivery and mode of delivery information

**Robson group**^a^	**Description**	**Total number of deliveries**	**Size of each category (%)**^**b**^	**Robson reference for size of each category**^**c**^	**Number of cesarean deliveries**	**Cesarean delivery frequency in each category (%)**^**d**^	**Robson guideline for achievable/ acceptable rate**^**e**^	**Contribution of each category to cesarean delivery rate (%)**^**f**^	**Contribution of each category to total cesarean deliveries (%)**^**g**^
1	Nulliparous women with a single cephalic pregnancy, ≥ 37 weeks gestation in spontaneous labor	446	20.9	35–42%	242	**54.3**	< 10%	11.3	24.9
2	Nulliparous women with a single cephalic pregnancy, ≥ 37 weeks gestation who either had labor induced or were delivered by cesarean delivery before labor	248	11.6		66	26.6	20–35%	3.1	6.8
3	Multiparous women without a previous uterine scar, with a single cephalic pregnancy, ≥ 37 weeks gestation in spontaneous labor	549	25.7	30%	158	**28.8**	Around 3%	7.4	16.3
4	Multiparous women without a previous uterine scar, with a single cephalic pregnancy, ≥ 37 weeks gestation who either had labor induced or were delivered by cesarean delivery before labor	336	15.7		47	14.0	< 15%	2.2	4.8
5	All multiparous women with at least one previous uterine scar, with a single cephalic pregnancy, ≥ 37 weeks gestation	296	13.9	‒	290	**98.0**	50–60%	13.6	29.9
6	All nulliparous women with a single breech pregnancy	26	1.2	3–4%	26	100.0	‒	1.2	2.7
7	All multiparous women with a single breech pregnancy, including women with previous uterine scars	38	1.8		38	100.0	‒	1.8	3.9
8	All women with multiple pregnancies, including women with previous uterine scars	21	1.0	1.5–2%	19	**90.5**	Around 60%	0.9	2.0
9	All women with a single pregnancy with a transverse or oblique lie, including women with previous uterine scars	5	0.2	‒	5	100.0	‒	0.2	0.5
10	All women with a single cephalic pregnancy < 37 weeks gestation, including women with previous scars	172	8.0	< 5%	79	**45.9**	Around 30%	3.7	8.1
Total		2137	100.0		970		‒	45.4	100.0

^a^World Health Organization guidelines for the Robson Classification advise that efforts to reduce cesarean delivery rates should be especially focused on reducing the rate in women in Robson group 1 (nulliparous women ≥ 37 weeks gestation singleton cephalic)

^b^Number of women in each category divided by the total number of women

^c^World Health Organization Robson Classification Implementation Manual reference guideline for assessing the type of population. Adapted from: World Health Organization. Robson Classification Implementation Manual. Available at: https://www.who.int/publications/i/item/9789241513197. Last accessed on June 27, 2023

^**d**^Number of cesarean deliveries in the category divided by the number of women in the category. Values that are higher than the Robson guideline for achievable/acceptable rates are bolded

^e^Adapted from: World Health Organization. Robson Classification Implementation Manual. Available at: https://www.who.int/publications/i/item/9789241513197. Last accessed on June 27, 2023

^f^Number of cesarean deliveries in each category divided by the total number of women

^g^Number of cesarean deliveries in each category divided by total number of cesarean deliveries

### Postpartum illnesses

Postpartum obstetric/gynecologic complications (e.g., postpartum hemorrhage, pulmonary embolus, death, seizure, and venous thromboembolism) were rare during the 6–8 weeks after delivery. Among women with cesarean deliveries, one woman reported vaginal discharge/infection. Among women with vaginal deliveries, only one woman reported persistent uterine bleeding and endometritis.

### Factors associated with cesarean delivery among women in Robson group 1

In this analysis, the final model was fitted without the 2- and 3-way interaction terms as none were found to be statistically significant. Among nulliparous women with singleton, cephalic, term pregnancies (Robson group 1), factors independently associated with cesarean delivery included age ≥ 25 years (adjusted odds ratio [aOR] 2.3, 95% confidence interval [CI] 1.5–3.4 for age 25–29 years; aOR 3.3, 95% CI 2.1–5.1 for age 30–34 years; aOR 5.6, 95% CI 3.4–9.2 for age ≥ 35 years), pre-pregnancy obesity (aOR 2.5, 95% CI 1.2–5.1), new or worsen medical condition during pregnancy (aOR 35.1, 95% CI 2.3–527.0), fetal distress (aOR 16.1, 95% CI 5.4–47.6), abnormal labor (aOR 6.3, 95% CI 1.7–24.2), infant size for gestational age ≥ 50^th^ percentiles (aOR > 2.0 for each decile starting from 50-59^th^ percentiles), and self-pay for delivery fees (aOR 1.7, 95% CI 1.1–2.8; Table 3). Women with existing blood conditions (e.g., sickle cell disease, thalassemia, or hemoglobinopathy) were less likely to have cesarean delivery (aOR 0.08, 95% CI 0.01–0.5).

**Table 3:** Risk factors for cesarean delivery among nulliparous women with singleton, cephalic, term pregnancies^a^

	Women with cesarean delivery (%)	Bivariate analysis		Multivariable analysis	
		Odds ratio (95% confidence interval)	*p*-value	Adjusted odds ratio (95% confidence interval)	*p*-value
**Age at delivery (year)**					
< 25	80 (27)	Reference		Reference	
25–29	107 (46)	2.3 (1.6–3.4)	< 0.01	2.3 (1.5–3.4)	< 0.01
30–34	91 (56)	3.5 (2.3–5.2)	< 0.01	3.3 (2.1–5.1)	< 0.01
≥ 35	87 (69)	6.2 (3.9–9.8)	< 0.01	5.6 (3.4–9.2)	< 0.01
**Pre-pregnancy body mass index**^**b**^					
Underweight	46 (31)	0.6 (0.4–0.9)	< 0.01	0.7 (0.5–1.1)	0.14
Normal	221 (44)	Reference		Reference	
Overweight	52 (53)	1.5 (0.9–2.2)	0.09	1.2 (0.7–2.0)	0.42
Obese	38 (73)	3.5 (1.9–6.6)	< 0.01	2.5 (1.2–5.1)	0.01
Unknown	8 (73)	3.4 (0.9–13.1)	0.07	2.7 (0.6–12.1)	0.20
**Existing blood condition**^c^					
No	363 (46)	Reference		Reference	
Yes	2 (8)	0.1 (0.02–0.5)	< 0.01	0.08 (0.01–0.5)	0.01
**New medical condition during the current pregnancy**^**b,**^ ^**d**^					
No	354 (44)	Reference		Reference	
Yes	11 (92)	14.0 (1.8–108.6)	0.01	35.1 (2.3–527.0)	0.01
**Fetal distress during current delivery**^**b**^					
No	331 (43)	Reference		Reference	
Yes	34 (89)	11.5 (4.0–32.6)	< 0.01	16.1 (5.4–47.6)	< 0.01
**Dysfunctional labor/failure to progress during current delivery**^**b**^					
No	349 (44)	Reference		Reference	
Yes	16 (84)	6.8 (2.0–23.6)	< 0.01	6.3 (1.7–24.2)	< 0.01
**Baby size for gestational age percentile based on INTERGROWTH-21st standard**^**b**^					
< 10^th^	28 (29)	Reference		Reference	
10-19^th^	35 (31)	1.1 (0.6–2.0)	0.74	1.2 (0.6–2.3)	0.65
20-29^th^	32 (36)	1.4 (0.7–2.6)	0.31	1.2 (0.6–2.5)	0.55
30-39^th^	35 (41)	1.7 (0.9–3.2)	0.09	1.5 (0.8–3.0)	0.24
40-49^th^	32 (39)	1.6 (0.9–3.0)	0.14	1.6 (0.8–3.2)	0.18
50-59^th^	40 (51)	2.6 (1.4–4.3)	< 0.01	2.3 (1.1–4.5)	0.02
60-69^th^	41 (48)	2.3 (1.3–4.3)	0.01	2.1 (1.1–4.1)	0.03
70-79th	41 (59)	3.7 (1.9–7.0)	< 0.01	2.8 (1.3–5.7)	< 0.01
80-89^th^	43 (69)	5.7 (2.8–11.3)	< 0.01	5.5 (2.6–11.8)	< 0.01
≥ 90^th^	37 (79)	9.2 (4.0–21.1)	< 0.01	6.9 (2.3–16.8)	< 0.01
Unknown	1 (25)	0.8 (0.1–8.6)	0.88	0.8 (0.1–8.2)	0.84
**Self-pay for delivery fees**					
Yes	65 (54)	1.5 (1.0–2.3)	0.03	1.7 (1.1–2.8)	0.02
No	300 (43)	Reference		Reference	

^a^Only women with gestational weeks at delivery and mode of delivery information were included; analyses taken into account the following items: characteristics of participants (age, nationality, marital status, employment, education, pre-pregnancy body mass index, insurance type, monthly household income, possession of household items, location of residence, use of cigarette and alcohol, and psychological stressor); delivery facility; year; prenatal care history (number of visits, trimester at first visit); illness history (type and number of existing chronic condition, type of new or worsen condition, hospitalization history); complication during current pregnancy (type and number of complication, trimester at diagnosis); and baby weight at delivery

^b^Information abstracted from medical records

^c^Blood problem such as sickle cell disease, thalassemia, hemoglobinopathy

^d^Questions asking about the following conditions: respiratory/lung problem; heart disease or heart condition; endocrine disorder such as thyroid problem or diabetes; blood problem such as sickle cell disease, or thalassemia; kidney or bladder disease; hepatitis, jaundice or liver disease, excluding hepatitis A and E; problem with the immune system, excluding HIV; cancer; neurologic or neuromuscular disorder; HIV infection; mental health condition such as anxiety or depression; and others

## Discussion

Among 2,137 pregnant women with gestational weeks at delivery and mode of delivery information who enrolled in a prospective cohort study, almost half of all women delivered babies by cesarean delivery. Based on Robson group size distribution, our study population was largely similar to the reference population in the WHO Robson Group Implementation Manual. However, our study population had higher cesarean delivery rates than those recommended for women with term, singleton, cephalic pregnancies including nulliparous women (Robson group 1), multiparous women without uterine scars in spontaneous labor (Robson group 3), and multiparous women with uterine scars (Robson group 5). Among nulliparous women with singleton, cephalic, term pregnancies (Robson group 1), factors associated with cesarean delivery were largely consistent with plausible indications for cesarean delivery with the exception of self-pay for delivery service.

The overall cesarean rate of 45% in this study is higher than the WHO’s estimates for Thailand from preceding years (31% in 2004–2008 global survey, 39% in 2010–2011 multi-country survey)[25], and other reports (32% in 2012, 31% in 2014, 42% in 2018) [12, 26, 27]. Our findings from 2017 and 2018 suggest that cesarean delivery rates in the study hospitals may be higher than other Thai hospitals, or rates may have increased since the latest published WHO estimates, consistent with other studies reporting increasing rates of cesarean delivery in Thailand [3]. We also found that out-of-pocket payment for delivery fees was associated with having cesarean delivery, suggesting that social and personal preferences may be a non-medical driver of elevated cesarean delivery rates. These findings are consistent with another study [28] among Thai mothers which also found that willingness to pay for delivery fees led to an increased likelihood of cesarean delivery. While our study did not examine factors contributing to patients’ preferences for cesarean delivery, a recent qualitative study among Thai women revealed that the reasons for such action were largely centered on the convenience of cesarean delivery to schedule birth, avoid labor pain, and ensure perceived safe childbirth [29].

When cesarean delivery rates are higher than expected, the WHO Robson Classification Implementation Manual recommends focusing efforts on rate reduction among nulliparous women with singleton, cephalic, term pregnancies (Robson group 1). Women in this group in fact represented about one fourth of all cesarean deliveries in our study, a rate much higher than the Robson achievable rate (< 10%) recommended by the WHO. Furthermore, cesarean delivery rates were higher than Robson guideline levels for multiparous women without a previous uterine scar, with a single cephalic pregnancy, ≥ 37 weeks gestation in spontaneous labor (Robson group 3) and multiparous women with ≥ 1 previous uterine scar, with a single cephalic pregnancy, ≥ 37 weeks gestation (Robson group 5), and women in these groups collectively accounted for 46% of all cesarean deliveries. Elevated cesarean delivery rate among multiparous women with uterine scars (Robson group 5) may reflect that VBAC has not been widely adopted in country. This may be explained by findings from a previous study in Thailand detailing findings from the country’s first institution-based VBAC program which reflected both an old belief “once a section always a section" [30] and the convenience and economic advantages of obstetricians [31]. In addition to support for VBAC with focus on high success rate and medical and economic advantages (e.g., lower hospital cost, shorter hospital stay, and reduced maternal and fetal morbidity and mortality), careful counseling of the patients, assessment of likelihood for successful VBAC delivery, and availability of appropriate facilities and staffing are needed for women considering this option for delivery [32, 33].

In this prospective cohort study of pregnant women, the detailed data collection from both participants' report and chart abstraction provided the opportunity to examine potential risk factors for cesarean delivery including maternal and fetal characteristics and obstetrical events that are not captured as part of the Robson Classification. The Robson Classification does not distinguish other potential obstetric indications for cesarean delivery, including delivery for maternal wellbeing (e.g., uncontrolled chronic hypertension or preeclampsia with severe features), obstetric indications (e.g., placental abruption, placenta previa), or fetal indications (e.g., fetal distress). Findings from this analysis suggest that such factors may influence obstetrical decision-making about mode of delivery in Thailand.

This study included a large sample of deliveries at two large tertiary level facilities that have some of the highest obstetric delivery rates in Thailand. A strength of this study was collection of data through both maternal report and from detailed review of medical records for prenatal and delivery information. However, limitations must be considered when interpreting study findings. First, this study was conducted during 2017 and 2018 precluding long-term assessment of changes in cesarean delivery rates and indications over time. Second, the study was conducted in tertiary level hospitals where rates of cesarean delivery may be higher than at other hospitals in Thailand because of the referral of complicated cases. Lastly, participants without gestational weeks at delivery and modes of delivery information differed from those with delivery mode confirmation with respect to selected baseline and pregnancy characteristics that may be associated with risk of cesarean delivery.

## Conclusions

At two high-volume tertiary obstetric hospitals in Bangkok, Thailand, cesarean delivery rate substantially exceeded the WHO-recommended target rate of 10–15%. Deliveries among three groups of women (nulliparous women with term, singleton cephalic pregnancies; multiparous women without uterine scars in spontaneous labor; and multiparous women with uterine scars) accounted for almost half of all cesarean deliveries. Our findings suggest that efforts to reduce non-clinically indicated cesarean delivery rates in Thailand might need to focus on reducing rates among multiparous women, in addition to nulliparous women with term, singleton cephalic pregnancies as generally recommended by the WHO Robson Classification Implementation Manual [34].

### Supplementary Information

**Additional file 1: Supplementary table 1.** Chronic medical condition and complication during the current pregnancy among those with gestational weeks at delivery and mode of delivery information who had ≥1 conditions. **Supplementary table 2.** Characteristics of participants (N [%]) with gestational weeks at delivery and mode of delivery information and included in the analysis versus those with missing gestational weeks at delivery and/or mode of delivery information and were excluded.

## Data Availability

The datasets used and/or analyzed during the current study are available from the corresponding author on reasonable request.

## Abbreviations

ANC: Antenatal Care
aOR: Adjusted Odds Ratio
BMI: Body Mass Index
CI: Confidence Interval
IRB: Institutional Review Board
MOPH: Ministry of Public Health
VBAC: Vaginal Birth After Cesarean Delivery
WHO: World Health Organization
