# Supplementary material for: Evaluation of cesarean delivery rates and factors associated with cesarean delivery among women enrolled in a pregnancy cohort study at two tertiary hospitals in Thailand
Source: BMC Pregnancy Childbirth. 2024 Feb 21;24:149. doi: 10.1186/s12884-024-06314-4 (PMC10880209; doi:10.1186/s12884-024-06314-4)
Supplement: Supplementary file 1 — Additional file 1: Supplementary table 1. Chronic medical condition and complication during the current pregnancy among those with gestational weeks at delivery and mode of delivery information who had ≥1 conditions. Supplementary table 2. Characteristics of participants (N [%]) with gestational weeks at delivery and mode of delivery information and included in the analysis versus those with missing gestational weeks at delivery and/or mode of delivery information and were excluded. [file 12884_2024_6314_MOESM1_ESM.docx]

**Supplementary table 1: Chronic medical condition and complication during the current pregnancy among those with gestational weeks at delivery and mode of delivery information who had ≥1 conditions.**

**A. Self-reported existing chronic medical condition^a^**

|  | **Total (N=271)**  **number (%)** | **Cesarean delivery (N=125)**  **number (%)** | **Vaginal delivery (N=146)**  **number (%)** |
| --- | --- | --- | --- |
| Endocrine condition | 79 (29) | 47 (38) | 32 (22) |
| Blood condition | 73 (27) | 10 (8) | 63 (43) |
| Heart condition | 44 (16) | 30 (24) | 14 (10) |
| Respiratory condition | 29 (11) | 17 (14) | 12 (8) |
| HIV infection | 3 (1) | 0 (0) | 3 (2) |
| Others^b^ | 69 (25) | 37 (30) | 32 (22) |

**B. Complication during the current pregnancy^a, c^**

|  | **Total (N=446)**  **number (%)** | **Cesarean delivery (N=253)**  **number (%)** | **Vaginal delivery (N=193)**  **number (%)** |
| --- | --- | --- | --- |
| Gestational diabetes | 267 (60) | 154 (61) | 113 (59) |
| Gestational hypertension | 134 (30) | 88 (35) | 46 (24) |
| Oligohydramnios | 22 (5) | 11 (4) | 11 (6) |
| Placenta previa | 18 (4) | 15 (6) | 3 (2) |
| Heavy uterine bleeding | 5 (1) | 3 (1) | 2 (1) |
| Severe anemia | 2 (<1) | 1 (<1) | 1 (<1) |
| Polyhydramnios | 1 (<1) | 0 (0) | 1 (<1) |
| Pre-eclampsia/ eclampsia | 2 (<1) | 2 (1) | 0 (0) |
| Incompetent cervix/cervical insufficiency | 1 (<1) | 0 (0) | 1 (<1) |
| Others^d^ | 53 (12) | 23 (9) | 30 (16) |

**^a^**Not mutually exclusive as some participants had >1 conditions**.**

^b^Participants with kidney/bladder condition; hepatitis, jaundice, liver disease; immunosuppressive condition not including HIV; cancer; neurologic/neuromuscular disease; mental health; tuberculosis; seizure; systemic lupus; rheumatoid arthritis; depression; and bipolar disorder.

**^c^**Information abstracted from medical records.

^d^Participants with intrauterine growth restriction; multiple myoma uteri; anterior vaginal cyst; uterine fibrosis; ovarian cyst; threaten abortion; premature rupture of membrane; urinary tract infection; chickenpox; anti-E positive; twin to twin transfusion; fetal beta thalassemia/hemoglobin E disease; and dead fetus in utero at 36 weeks.

**Supplementary table 2: Characteristics of participants (N [%]) with gestational weeks at delivery and mode of delivery information and included in the analysis versus those with missing gestational weeks at delivery and/or mode of delivery information and were excluded.**

| **Characteristic** | **All participants** | | | **Only participants with available information on characteristic of interest** | | |
| --- | --- | --- | --- | --- | --- | --- |
|  | **Participants with gestational weeks at delivery and mode of delivery information** | **Participants missing gestational weeks at delivery and/or mode of delivery information** | **P-value^a^** | **Participants with gestational weeks at delivery and mode of delivery information** | **Participants missing gestational weeks at delivery and mode of delivery information** | **P-value^b^** |
| **Age at delivery (years)** | N=2137 | N=673 | <0.01 | N=2137 | N=214 | 0.19 |
| 25 | 513 (24) | 55 (8) |  | 513 (24) | 55 (8) |  |
| 25-29 | 557 (26) | 67 (10) |  | 557 (26) | 67 (10) |  |
| 30-34 | 530 (25) | 50 (7) |  | 530 (25) | 50 (7) |  |
| ≥35 | 537 (25) | 42 (6) |  | 537 (25) | 42 (6) |  |
| Unknown | 0 (0) | 459 (68) |  | - | - |  |
| **Marital status** | N=2137 | N=673 | 0.23 | N=2135 | N=673 | 0.12 |
| Married or cohabitating | 2066 (97) | 659 (98) |  | 2066 (97) | 659 (98) |  |
| Single, divorced, widowed, separated | 69 (3) | 14 (2) |  | 69 (3) | 14 (2) |  |
| Unknown | 2 (<1) | 0 (0) |  | - | - |  |
| **Highest year of school completed** | N=2137 | N=673 | <0.01 | N=2130 | N=672 | <0.01 |
| None | 61 (3) | 19 (3) |  | 61 (3) | 19 (3) |  |
| Primary school | 771 (36) | 293 (44) |  | 771 (36) | 293 (44) |  |
| Secondary | 533 (25) | 158 (23) |  | 533 (25) | 158 (23) |  |
| Post-secondary/university | 765 (36) | 202 (30) |  | 765 (36) | 202 (30) |  |
| Unknown | 7 (<1) | 1 (<1) |  | - | - |  |
| **Monthly household income (Baht)** | N=2137 | N=673 | 0.34 | N=2133 | N=671 | 0.28 |
| <20000 | 573 (27) | 201 (30) |  | 573 (27) | 201 (30) |  |
| 20000-39999 | 993 (46) | 313 (47) |  | 993 (46) | 313 (47) |  |
| 40000-49999 | 231 (11) | 62 (9) |  | 231 (11) | 62 (9) |  |
| ≥50000 | 336 (16) | 95 (14) |  | 336 (16) | 95 (14) |  |
| Unknown | 4 (<1) | 2 (<1) |  | - | - |  |
| **Type of health insurance used for baby delivery** | N=2137 | N=673 | <0.01 | N=2135 | N=350 | <0.01 |
| Social Security Scheme | 1029 (48) | 197 (29) |  | 1029 (48) | 197 (29) |  |
| Universal Coverage Scheme | 398 (19) | 73 (11) |  | 398 (19) | 73 (11) |  |
| Civil Servant Medical Benefit Scheme (including state enterprises’) | 109 (5) | 22 (3) |  | 109 (5) | 22 (3) |  |
| Health card | 226 (11) | 19 (3) |  | 226 (11) | 19 (3) |  |
| No insurance (self-pay) | 342 (16) | 36 (5) |  | 342 (16) | 36 (5) |  |
| Others^c^ | 31 (1) | 3 (<1) |  | 31 (1) | 3 (<1) |  |
| Unknown | 2 (<1) | 323 (48) |  | - | - |  |
| **Pre-pregnancy body mass index (kg/m^2^)^d^** | N=2137 | N=673 | 0.14 | N=2111 | N=661 | 0.13 |
| <18.5 (underweight) | 319 (15) | 119 (18) |  | 319 (15) | 119 (18) |  |
| 18.5-24.9 (normal) | 1271 (59) | 378 (56) |  | 1271 (59) | 378 (56) |  |
| 25-29.9 (overweight) | 336 (16) | 116 (7) |  | 336 (16) | 116 (7) |  |
| ≥30 (obese) | 185 (9) | 48 (7) |  | 185 (9) | 48 (7) |  |
| Unknown | 26 (1) | 12 (2) |  | - | - |  |
| **Parity^d^** | N=2137 | N=673 | 0.31 | N=2300 | N=510 | 0.31 |
| 0 | 930 (44) | 308 (46) |  | 930 (44) | 308 (46) |  |
| ≥1 | 1207 (56) | 365 (54) |  | 1207 (56) | 365 (54) |  |
| **Self-reported number of current chronic medical condition^e^** | N=2137 | N=673 | <0.01 | N=2137 | N=673 | <0.01 |
| 0 | 1866 (87) | 625 (93) |  | 1866 (87) | 625 (93) |  |
| 1 | 245 (11) | 44 (7) |  | 245 (11) | 44 (7) |  |
| >1 | 26 (1) | 4 (1) |  | 26 (1) | 4 (1) |  |
| **New underlying medical condition during the current pregnancy^d^** | N=2137 | N=673 | 0.01 | N=2137 | N=673 | 0.01 |
| Yes | 43 (2) | 4 (1) |  | 43 (2) | 4 (1) |  |
| No | 2094 (98) | 669 (99) |  | 2094 (98) | 669 (99) |  |
| **Worsening chronic medical condition during the current pregnancy^d^** | N=2137 | N=673 | 0.79 | N=2137 | N=673 | 0.79 |
| Yes | 14 (1) | 5 (1) |  | 14 (1) | 5 (1) |  |
| No | 2123 (99) | 668 (99) |  | 2123 (99) | 668 (99) |  |
| **Psychosocial stressor**^f^ | N=2137 | N=673 | <0.01 | N=2135 | N=376 | 0.11 |
| Yes | 336 (16) | 47 (7) |  | 336 (16) | 47 (7) |  |
| No | 1799 (84) | 329 (49) |  | 1799 (84) | 329 (49) |  |
| Unknown | 2 (<1) | 297 (44) |  | - | - |  |
| **Previous uterine scar^d^** | N=2137 | N=673 | <0.01 | N=2137 | N=673 | <0.01 |
| Yes | 343 (16) | 60 (9) |  | 343 (16) | 60 (9) |  |
| No | 1794 (84) | 613 (91) |  | 1794 (84) | 613 (91) |  |
| **Multiparous^d^** | N=2137 | N=673 | <0.01 | N=2137 | N=673 | <0.01 |
| Yes | 21 (1) | 407 (60) |  | 21 (1) | 407 (60) |  |
| No | 2116 (99) | 266 (40) |  | 2116 (99) | 266 (40) |  |
| **Number of prenatal care visits before delivery^d^** | N=2137 | N=673 | <0.01 | N=2124 | N=347 | 0.28 |
| 1-3 | 43 (2) | 12 (2) |  | 43 (2) | 12 (2) |  |
| 4-6 | 170 (8) | 29 (4) |  | 170 (8) | 29 (4) |  |
| 7-9 | 775 (36) | 115 (17) |  | 775 (36) | 115 (17) |  |
| >9 | 1136 (53) | 191 (28) |  | 1136 (53) | 191 (28) |  |
| Unknown | 13 (1) | 326 (48) |  | - | - |  |
| **Trimester of 1^st^ prenatal care visit^d^** | N=2137 | N=673 | <0.01 | N=2133 | N=556 | 0.04 |
| First | 1477 (70) | 414 (62) |  | 1477 (70) | 414 (62) |  |
| Second | 622 (29) | 137 (20) |  | 622 (29) | 137 (20) |  |
| Third | 34 (2) | 5 (1) |  | 34 (2) | 5 (1) |  |
| Unknown | 4 (<1) | 117 (17) |  | - | - |  |
| **Number of complications during this pregnancy^d, g^** | N=2137 | N=673 | <0.01 | N=2300 | N=510 | <0.01 |
| 0 | 1691 (79) | 620 (92) |  | 1691 (79) | 620 (92) |  |
| 1 | 392 (18) | 46 (7) |  | 392 (18) | 46 (7) |  |
| >1 | 54 (3) | 7 (1) |  | 54 (3) | 7 (1) |  |
| **Infant weight at delivery^d^** | N=2137 | N=673 | <0.01 | N=2137 | N=673 | <0.01 |
| Small for gestational age^h^ | 194 (9) | 12 (2) |  | 194 (9) | 12 (2) |  |
| Normal | 1769 (83) | 651 (97) |  | 1769 (83) | 651 (97) |  |
| Large for gestational age^h^ | 174 (8) | 10 (1) |  | 174 (8) | 10 (1) |  |
| **Presentation^d^** | N=2137 | N=673 | <0.01 | N=2137 | N=165 | 0.05 |
| Cephalic | 2040 (95) | 152 (23) |  | 2040 (95) | 152 (23) |  |
| Breech | 68 (3) | 9 (1) |  | 68 (3) | 9 (1) |  |
| Transverse | 5 (<1) | 2 (<1) |  | 5 (<1) | 2 (<1) |  |
| Other malpresentation | 24 (1) | 2 (<1) |  | 24 (1) | 2 (<1) |  |
| Unknown | 0 (0) | 508 (75) |  | - | - |  |
| **Onset of labor^d^** | N=2137 | N=673 | <0.01 | N=2137 | N=673 | <0.01 |
| Spontaneous | 1509 (71) | 279 (41) |  | 1509 (71) | 279 (41) |  |
| Induction^i^ | 628 (29) | 394 (59) |  | 628 (29) | 394 (59) |  |
| **Cesarean delivery planned** | N=2137 | N=673 | <0.01 | N=2137 | N=673 | <0.01 |
| Yes | 807 (38) | 86 (13) |  | 807 (38) | 86 (13) |  |
| No | 1330 (62) | 587 (87) |  | 1330 (62) | 587 (87) |  |
| **Gestational weeks at delivery^d^** | N=2137 | N=673 | <0.01 | N=2137 | N=208 | 0.02 |
| <37 | 199 (9) | 31 (5) |  | 200 (9) | 30 (15) |  |
| 37-38 | 875 (41) | 67 (10) |  | 876 (41) | 66 (32) |  |
| 39-40 | 995 (47) | 104 (15) |  | 995 (47) | 104 (50) |  |
| ≥41 | 68 (3) | 6 (1) |  | 68 (3) | 6 (3) |  |
| Unknown | 0 (0) | 465 (69) |  | - | - |  |
| **Length of hospital stay (days)^d^** | N=2137 | N=673 | <0.01 | N=2060 | N=165 | <0.01 |
| <3 | 458 (21) | 91 (14) |  | 458 (21) | 91 (14) |  |
| 3 | 820 (38) | 50 (7) |  | 820 (38) | 50 (7) |  |
| 4-6 | 639 (30) | 18 (3) |  | 639 (30) | 18 (3) |  |
| 7-13 | 91 (4) | 2 (<1) |  | 91 (4) | 2 (<1) |  |
| ≥14 | 52 (2) | 4 (1) |  | 52 (2) | 4 (1) |  |
| Unknown | 77 (4) | 508 (75) |  | - | - |  |

^a^Comparing all participants in two groups including those with missing information on characteristic of interest.

^b^Comparing participants in two groups excluding those with missing information on characteristic of interest.

^c^Private health insurance, handicap card, and others.

^d^Information abstracted from medical records.

^e^Questions asking about the following conditions: respiratory/lung problems; heart disease or heart condition; endocrine disorders such as thyroid problems or diabetes; blood problem such as sickle cell disease, or thalassemia; kidney or bladder disease; hepatitis, jaundice or liver disease, excluding hepatitis A and E; problems with the immune system, excluding HIV; cancer; neurologic or neuromuscular disorder; HIV infection; mental health condition such as anxiety or depression; and others.

^f^Based on 17 questions about major events or changes that may have happened to one’s life.

^g^Questions asking about the following conditions: gestational diabetes, gestational hypertension, oligohydramnios, placenta previa, heavy uterine bleeding, severe anemia, polyhydramnios, pre-eclampsia, eclampsia, incompetent cervix or cervical insufficiency, intrauterine growth restriction, multiple myoma uteri, anterior vaginal cyst, uterine fibrosis, ovarian cyst, threatened abortion, premature rupture of membrane, urinary tract infection, chickenpox, anti-E positive, twin to twin transfusion, fetal beta thalassemia/hemoglobin E disease, and dead fetus in utero at 36 weeks.

^h^Small for gestational age was defined as birth weight <10% of the same gestational age and gender in the same population; large for gestational age was defined as birth weight >90% of the same gestational age and gender in the same population.

^i^All women with gestational weeks >40 were induced.
